# Inhibition of Nrf2/HO-1 signaling pathway by Dextran Sulfate suppresses angiogenesis of Gastric Cancer

**DOI:** 10.7150/jca.50605

**Published:** 2021-01-01

**Authors:** Yuanyi Xu, Yuanyuan Yang, Yunning Huang, Qian Ma, Jing Shang, Jiaxin Guo, Xiangmei Cao, Xiaofei Wang, Mengqi Li

**Affiliations:** 1 Department of Pathology, Ningxia Medical University, Yinchuan, Ningxia 750004, China.; 2 Department of Gastrointestinal Surgery, The Affiliated People's Hospital of Ningxia Medical University, Yinchuan, Ningxia 750001, China.; 3 College of Life Sciences, Ningxia University, Yinchuan, Ningxia 750021, China.; 4 College of Basic Medicine, Ningxia Medical University, Yinchuan, Ningxia 750004, China.; 5 Third Clinical Medical College, Ningxia Medical University, Yinchuan, Ningxia 750004, China.; 6 Department of Pathology, North China University of Science and Technology Affiliated Hospital, Tangshan, Hebei 063000, China.

**Keywords:** dextran sulfate, gastric cancer, metastasis, angiogenesis, Nrf2/HO-1, VEGF

## Abstract

**Purpose:** To investigate the role of Nrf2/HO-1 signaling pathway in angiogenesis and whether dextran sulfate (DS) could suppress angiogenesis by inhibiting Nrf2/HO-1 signaling pathway in gastric cancer.

**Methods:** *In vitro;* Western blot analyzed the expression of Nrf2 in gastric cell lines. Tube formation assay observed the effect of gradient concentration DS on the angiogenic potential of HGC-27 cells. Immunofluorescence,western blot and qPCR analyzed the effects of DS on the expression of Nrf2, HO-1 and VEGF under gradient hypoxia time. Immunofluorescence,western blot,qPCR and tube formation assay analyzed the effects of up-regulating or down-regulating Nrf2/HO-1 signaling pathway on VEGF expression and angiogenic potential in HGC-27 cells. *In vivo:* Construct nude mouse intraperitoneal implantation metastasis model. Immunohistochemistry and western blot analyzed the effects of DS on the expression of Nrf2, HO-1, VEGF and MVD in nude mice. Immunohistochemistry detected the expression of Nrf2, HO-1, VEGF and MVD in human paracancerous tissue and gastric cancer tissues with different degrees of differentiation.

**Results:** The expression of Nrf2 increased most significantly in HGC-27 cell line. DS reduced the angiogenic potential and the expression of Nrf2, HO-1 and VEGF in HGC-27 cells. Down-regulation of Nrf2/HO-1 signaling pathway decreased VEGF expression and angiogenic potential in HGC-27 cells. Up-regulation of Nrf2/HO-1 signaling pathway increased VEGF expression and angiogenic potential in HGC-27 cells. DS reduced the expression of Nrf2, HO-1, VEGF and MVD in nude mice. Nrf2, HO-1, VEGF and MVD showed low expression in paracancerous tissue but high expression in gastric cancer tissues. They were weak, moderate and strong in well, moderately and poorly differentiated gastric cancer tissues, respectively.

**Conclusion:** Nrf2/HO-1 signaling pathway may positively regulate gastric cancer angiogenesis and DS may suppress the angiogenesis by inhibiting Nrf2/HO-1 signaling pathway in gastric cancer.

## Introduction

Gastric cancer is the fifth most common cancer and the third leading cause of cancer-related mortality in the world 1. Metastasis is the leading cause of death in patients with gastric cancer 2. Gastric cancer metastasis is a complex process involving numerous factors and multiple stages, and angiogenesis plays a central role in the growth and spread of tumor cells 3. It not only provides necessary nutrients for tumor cells, removes metabolites produced by tumor cell growth, but also provides channels for distant metastasis of tumor cells, enabling the tumor to acquire the ability to further grow and metastasize. Many angiogenic factors are involved in the complex regulation of angiogenesis. Tumor angiogenesis and its associated factors are highly relevant targets for controlling the metastasis of gastric cancer. Consequently, pharmacologic blockade of angiogenesis by inhibiting these angiogenic factors is a promising strategy for surpressing gastric cancer metastasis.

Traditionally considered as the master regulator of cytoprotective responses against exogenous or endogenous stressors, the signal pathway formed by transcription factor nuclear factor-erythroid 2-related factor 2 (Nrf2) and its downstream target gene heme oxygenase-1 (HO-1) has recently been found to promote cancer development, malignant progression, therapy resistance and poor prognosis 4. In the last years, Nrf2 has emerged as a promising target in cancer treatment and some studies have been made to identify therapeutic strategies aimed at disrupting its prooncogenic role. Importantly, the aberrant activation or accumulation of Nrf2 is a common event in many tumors 5, especially in solid tumors that are prone to stress response due to the hypoxic microenvironment, such as gastric cancer 6. It has been found that the expression of Nrf2 in gastric cancer is a potential indicator of poor prognosis 7. However, the role of the Nrf2/HO-1 signaling pathway in gastric cancer, especially on angiogenesis, remains unclear. It suggests that Nrf2/HO-1 signaling pathway may be potential molecular targets for anti-angiogenic treatment of gastric cancer.

Abdominal metastasis is the most common metastasis method for gastric cancer 8. Chemotherapy has become the main treatment for abdominal metastasis of gastric cancer. Most gastric cancer intraperitoneal chemotherapy drugs cannot achieve long-term effective drug concentration due to their rapid absorption in the abdominal cavity 9. Not only that, they are trapped by the limitations of drug resistance and side effects. Dextran sulfate (DS) used in this study is a macromolecular dextran derivative with a relative molecular mass of 5 × 10^5^. It has advantages of slow absorption, long action time and little side effects in the abdominal cavity 10. In a previous study, our research group found that DS could reduce the number and volume of tumor nodules in nude mouse intraperitoneal implantation metastasis model 10. It suggests that DS may suppress gastric cancer abdominal metastasis by inhibiting angiogenesis. However, the specific molecular mechanism has not been fully defined.

In the present study, we investigated the role of Nrf2/HO-1 signaling pathway in angiogenesis of gastric cancer and whether DS could decrease vascular endothelial growth factor (VEGF) expression to suppress angiogenesis by down-regulating Nrf2/HO-1 signaling pathway in gastric cancer under hypoxic microenvironment.

## Materials and methods

### Drugs and formulas

DS, dimethyl sulphoxide (DMSO) and tert-butylhydroquinone (tBHQ) were obtained from Sigma-Aldrich (St. Louis, MO, US). Weigh an appropriate amount of DS and dissolve it in phosphate buffered saline (PBS). Sterilize it with a 22 μm pore-size syringe filter after mixing and dilute to the required concentration with culture medium before use. Brusatol (BRU) was obtained from Tauto (Shanghai, China). Weigh an appropriate amount of BRU or tBHQ and dissolve it in DMSO (0.1% DMSO final concentration). Sterilize it with a 22 μm pore-size syringe filter after mixing and dilute to the required concentration with culture medium before use.

### Antibodies and primers

The antibodies against Nrf2 (ab62352), HO-1 (ab13248), VEGF (ab46152) and CD34 (ab81289) were obtained from Abcam (Cambridge, MA, USA). The antibodies against β-Tubulin and GAPDH were obtained from Cwbio (Beijing, China). The primers of Nrf2, HO⁃1, VEGF and GAPDH were synthesized by Sangon (Shanghai, China).

### Cell lines, nude mice and human specimens

This study was approved by the Medical Ethics Review Committee of Ningxia Medical University (Yinchuan, China; registration no. 2019-054).

Human undifferentiated gastric cancer cell line HGC-27 was donated by Harbin Medical University. Human poorly differentiation gastric cancer cell line MKN-45,well differentiation gastric cancer cell line AGS and normal gastric mucosal epithelial cell line GES-1 were donated from East China Normal University. Human umbilical vein endothelial cell line HUVEC was donated by the General Hospital of Ningxia Medical University.

BALB/c nude mice were obtained from Vitalriver (Beijing, China). The animal license number is SCXX (Beijing) 2006-0009. Nude mice were male, 5 to 6 weeks old, and 18 to 22g body weight. The animals were raised under SPF conditions and had free access to sterile food and water. All animal experiments conformed to the institutional animal care and use guidelines.

All of the human samples were obtained with informed consent from all individuals participating in the present study. Paracancerous tissue and gastric cancer tissues were surgically resected and diagnosed as gastric cancer by pathological examination in the Affiliated People's Hospital of Ningxia Medical University. The paracancerous tissue to the cancer was more than 5 cm away from the cancerous site and no cancerous tissue was found by pathological examination. Gastric cancer tissues were classified into well, moderately, poorly differentiated according to pathological stage.

### Cell culture

HGC cells were maintained in RPMI-1640 medium supplemented with 10% FBS and 1% Penicillin-Streptomycin at 37 °C in the presence of 5% CO_2_ in a humidified incubator. HUVEC cells were maintained in DMEM/high glucose medium supplemented with 10% FBS and 1% Penicillin-Streptomycin at 37 °C in the presence of 5% CO_2_ in a humidified incubator.

### Tube formation assay

HGC-27 cells culture supernatant of each group was collected for later use. 50 μl Matrigel (Corning, Bedford, MA, USA) was added to each well in a 96-well plate. 2 × 10^4^ HUVEC cells per well was seeded on 96-well plates and 100 μl HGC-27 cells culture supernatant was added to each group. HUVEC cells were cultivated at 37°C in the presence of 1% O_2_ and 5% CO_2_ in a humidified incubator to form capillary-like structures. Five fields of view were selected randomly at 100× magnification by Image J software to obtain the number of tube formation in each group.

### Immunofluorescence

HGC-27 cells grown on glass cover slips were fixed with 4% paraformaldehyde for 15 minutes, permeated with 0.3% TritonX-100 for 20 minutes, and blocked with 10% goat serum for 30 minutes. The cells were incubated with the primary antibody overnight at 4 °C and PBS replaced the primary antibody as a negative control. After washing with PBS, the cells were incubated with the secondary antibody at 37 °C for 30 minutes. Cover slips were covered with fluorescent mounting medium with DAPI. Nrf2 and VEGF were labeled with FITC, HO-1 was labeled with rhodamine, and cell nucleus was labeled with DAPI. Five fields of view were selected randomly at 400× magnification by Image-Pro Plus software to obtain the mean optical density (MOD) of the view.

### Western blot

Total protein of each group was extracted using Whole Cell Lysis Assay (KeyGEN, Jiangsu, China) following the manufacturer's instructions. The concentration of protein was detected using BCA Protein Quantitation Assay (KeyGEN, Jiangsu, China) according to the manufacturer's instructions. First,the proteins were separated in a sodium dodecyl sulfate-polyacrylamide gel electrophoresis (SDS-PAGE) and transferred onto polyvinylidene difluoride (PVDF) membranes. Then, they were incubated with the indicated primary antibodies and corresponding horse radish peroxidase-conjugated secondary antibodies. Last, the detection is performed by enhanced chemiluminescence (ECL). β-Tubulin and GAPDH were used as loading controls. Image J software measured the gray value of the band and the ratio of gray value was taken as the relative expression of protein.

### qPCR

Total RNA of each group was extracted using E.Z.N.A™. Total RNA Kit I (Omega Bio-Tek, Norcross, GA, USA) following the manufacturer's instructions. RNA was used as a template to synthesis of cDNA in a reverse transcription reaction by PrimeScript™ RT Master Mix (TaKaRa, Kyoto, Japan) according to the manufacturer's instructions. Real Time PCR reaction was performed by TB Green™ Premix Ex Taq™ II (TaKaRa, Kyoto, Japan) in accordance with the manufacturer's instructions. GAPDH was used as a loading control. The relative expression level of mRNA was calculated by the 2^-△CT^ method. The primer sequences are as follows:

- Nrf2, Forward: 5′-AACACAAGAGCCCCTGTGTGGC-3′, Reverse: 5′-TGCCCCTGAGATGGTGACAA-3′;
- HO-1, Forward: 5′-GGCCTCCCTGTACCACATCT-3′, Reverse: 5′-CTGCATGGCTGGTGTGTAGG-3′;
- VEGF, Forward: 5′-GCCATCCAATCGAGACCCTG -3′, Reverse: 5′-GGCACACAGGATGGCTTGAA -3′;
- GAPDH, Forward: 5′-CAGGAGGCATTGCTGATGAT-3′, Reverse: 5′-GAAGGCTGGGGCTCATTT-3′.

### Construct nude mouse intraperitoneal implantation metastasis model

Thirty nude mice were randomly divided into Control group and DS group, and fifteen mice in each group. Each nude mouse was intraperitoneally injected with 5 × 10^7^ cells/ml cell suspension (0.2 ml/head). After 24 hours, the Control group was injected with saline (1 ml/head) and the DS group was injected with 0.3% DS (1 ml/head).The whole process strictly followed aseptic operation. The nude mice were sacrificed by cervical dislocation on 14th days. In the tumor model constructed in this study, the tumor load did not exceed 5% of the body weight of nude mice and the tumor diameter did not exceed 15 mm. The omentum tissue implanted with tumor nodules was used for subsequent experiments.

### Immunochemistry

The samples were fixed, embedded and sliced (4 μm). Conventionally baked, dewaxed and hydrated. Antigen repaired in sodium citrate buffer. 3% H_2_O_2_ blocked endogenous peroxidase activity. Goat serum blocked non-specific sites. The samples were incubated with primary antibody at 4 °C overnight. PBS replaced the primary antibody as a negative control. After washing with PBS, the samples were incubated with secondary antibody at 37 °C for 30 minutes. The following steps were performed: color development, counterstaining, differentiation, dehydration, and transparency. Finally, the samples fixed with neutral resin. Five fields of view were selected randomly by Image-Pro Plus software to obtain the mean optical density (MOD) of the view.

### MVD count

According to the Weidner counting method, the area of dense microvessels were found under a low magnification lens, then the number of microvessels in 5 fields of view were counted with a high magnification lens, and the average value was used as the microvessel density (MVD) value. Any vascular endothelial cells or endothelial cell clusters that could be stained brown-yellow, and there was a clear separation between adjacent blood vessels, tumor cells or other tissues were regarded as a microvessel.

### Statistical analysis

Statistical analyses were performed using the SPSS23.0 statistical analysis software. Figures were constructed using the GraphPad Prism 8.0 software. Data were presented as the mean ± standard deviation and *P* < 0.05 was considered as statistically significant. The comparison of two sample means was performed using independent sample t test. All experiments were repeated three times independently.

## Results

### *In vitro* results

#### The expression of Nrf2 in gastric cell lines

**Figure 1:**
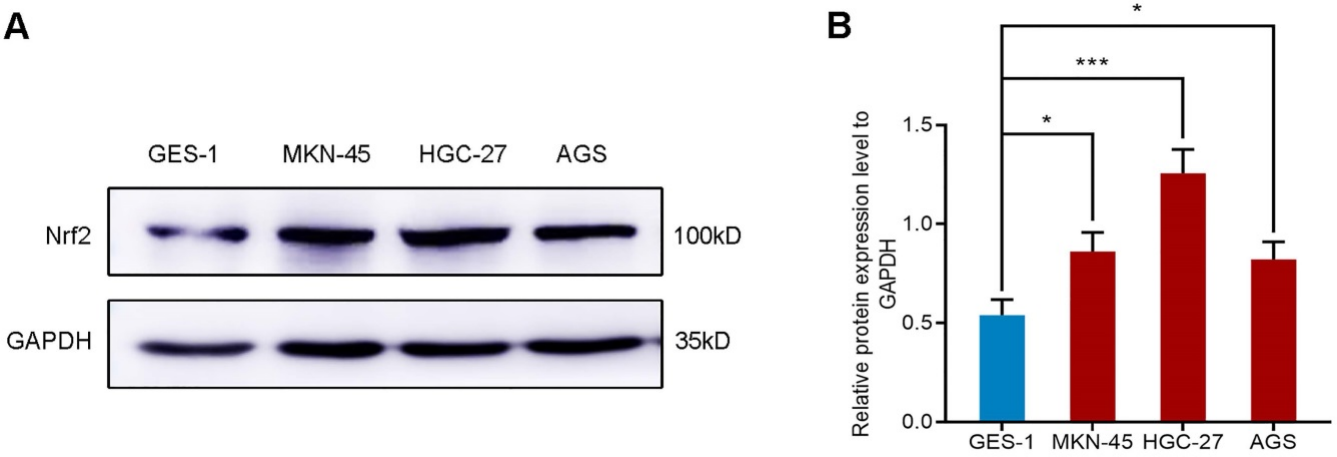
Western blot analyzes the protein expression of Nrf2 in different gastric cell lines. * *P* <0.05, *** *P* < 0.001.

We selected four different gastric cell lines and observed the expression of Nrf2 in them. The results of western blot showed that the expression level of Nrf2 in gastric cancer cell lines AGS, MKN-45 and HGC-27 was higher than that of normal gastric mucosal epithelial cell line GES-1 (**Figure 1**). In particular, the expression of Nrf2 increased most significantly in the HGC-27 cell line. Consequently, we selected the HGC-27 cell line as the research object for subsequent research.

### DS inhibits the angiogenic potential of HGC-27 cells

**Figure 2:**
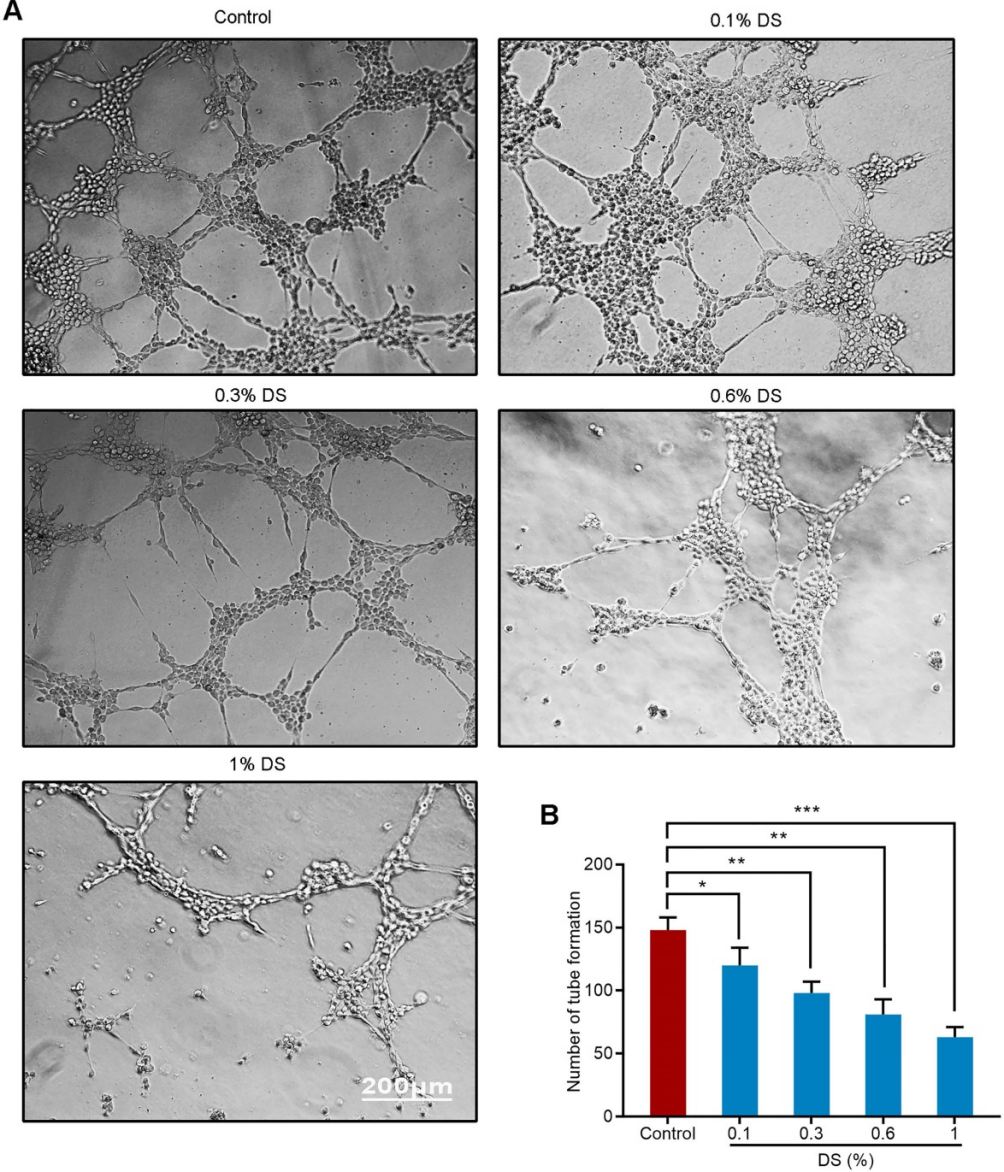
Tube formation assay shows the effect of gradient concentration DS on the angiogenic potential of HGC-27 cells. HGC-27 cells were treated with a gradient concentration of DS at 0%, 0.1%, 0.3%, 0.6%, 1% under hypoxic conditions, accordingly. Scale bar, 200 µm. * *P* <0.05, ** *P* <0.01, *** *P* < 0.001.

We first evaluated the effect of gradient concentration DS (0%, 0.1%, 0.3%, 0.6%, 1%) on the ability of HGC-27 cells to induce angiogenesis under hypoxic microenvironment (**Figure 2**). The results of tube formation assay showed that the number of tube formation in the Control group was 148 ± 10, and the number of tube formation in the gradient concentration DS (0.1%, 0.3%, 0.6%, 1%) groups were 120 ± 14, 98 ± 9, 81 ± 12,63 ± 8, respectively. Compared with Control group, the reductions of tube formation in different concentrations of DS (0.1%, 0.3%, 0.6%, 1%) groups were 18.9%, 33.8%, 45.3% and 57.4%, accordingly. With the increase of DS concentration, the number of tube formation gradually decreased and the capillary-like structures tended to break. It indicates that DS can inhibit the ability of human gastric cancer HGC-27 cells to induce angiogenesis and this effect is concentration-dependent.

#### DS reduces the expression of Nrf2, HO-1 and VEGF in HGC-27 cells

**Figure 3:**
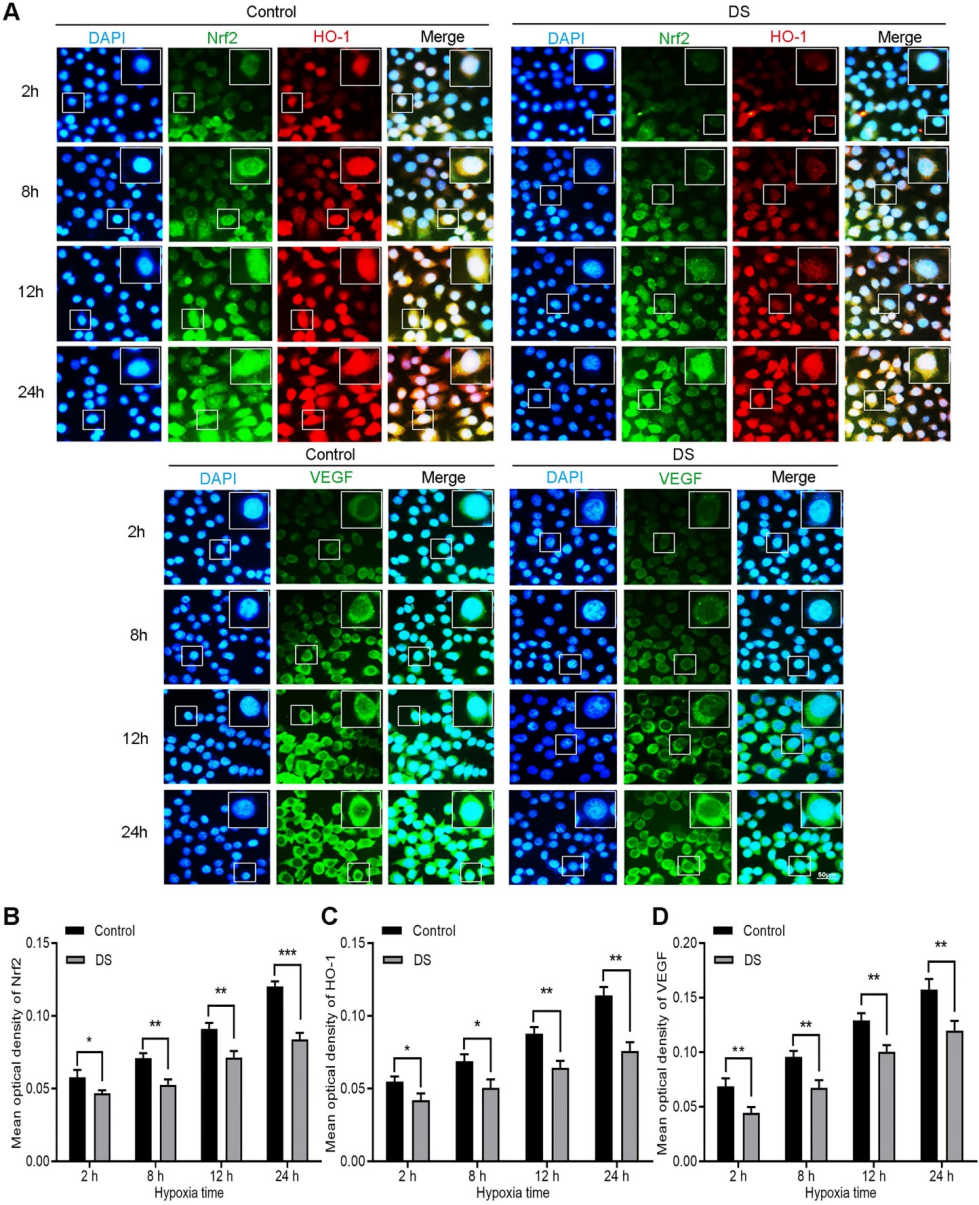
Immunofluorescence observes the effects of DS on the expression of Nrf2, HO-1 and VEGF in HGC-27 cells under gradient hypoxia time.Nrf2 and VEGF are labeled with FITC (green) while HO-1 is labeled with Rhodamine (red). Scale bar, 50 µm. * *P* <0.05, ** *P* <0.01, *** *P* < 0.001.

Immunofluorescence observed the expression of Nrf2, HO-1 and VEGF in HGC-27 cells after culturing for 2, 8, 12 and 24 hours under hypoxic microenvironment (**Figure 3**). Nrf2 and HO-1 were labeled by immunofluorescence double staining. The results showed that Nrf2 and HO-1 were mainly distributed in the cytoplasm and nucleus. At the same time, we observed the co-localization of Nrf2 and HO-1 was concentrated in the cytoplasm and nucleus. VEGF was mainly distributed in the cell membrane and cytoplasm. The fluorescence intensities of Nrf2, HO-1 and VEGF in the DS group was significantly reduced than that in the Control group and the differences were statistically significant at different time points (*P* <0.05). In addition, the fluorescence intensities of Nrf2, HO-1 and VEGF increased gradually with the prolongation of hypoxic culture time. These findings indicate that DS reduces the expression of Nrf2, HO-1 and VEGF in human gastric cancer HGC-27 cells.

**Figure 4:**
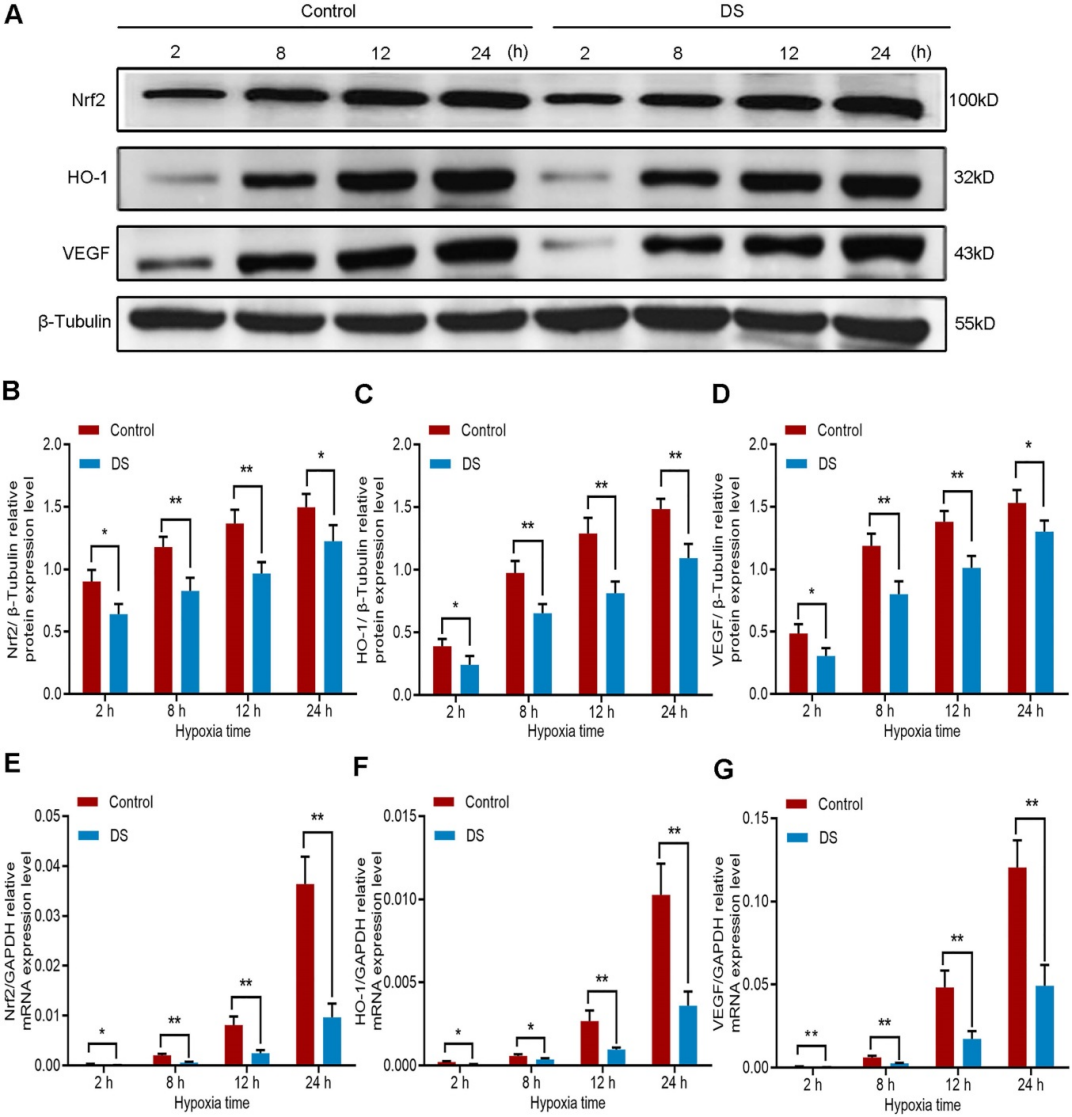
Western blot (A-D) and qPCR (E-F) analyze the effects of DS on Nrf2, HO-1 and VEGF at protein and mRNA expression levels in HGC-27 cells under gradient hypoxia time. * *P* <0.05, ** *P* <0.01.

Furthermore, we detected the protein expression of Nrf2, HO-1 and VEGF in HGC-27 cells under the same conditions. The results of western blot (**Figure 4A-D**) showed that the protein expression of Nrf2, HO-1 and VEGF gradually increased with the prolongation of hypoxic culture time, especially at 12 and 24 hours. At the same time point, the expression of DS group was significantly lower than that of Control group. To determine whether the decrease in Nrf2, HO-1 and VEGF protein levels inhibited by DS could be attributable to a decrease in transcription, we then assessed Nrf2, HO-1 and VEGF mRNA levels by qPCR (**Figure 4E-G**). The changes in protein level of the three were consistent with the changes in mRNA level. Collectively, these results demonstrate that the protein and mRNA expression of Nrf2, HO-1 and VEGF increased gradually with prolonged hypoxia time and DS can inhibit the protein and mRNA expression of Nrf2, HO-1 and VEGF in human gastric cancer HGC-27 cells.

#### Down-regulation of Nrf2/HO-1 signaling pathway decreased VEGF expression and angiogenic potential in HGC-27 cells

We introduced the Nrf2 inhibitor BRU in order to further explore the role of Nrf2/HO-1 signaling pathway in the angiogenesis of human gastric cancer cells. Firstly, western blot detected the protein expression of Nrf2, HO-1 and VEGF with a gradient concentration of BRU (0 nM, 10 nM, 20 nM, 40 nM, 80 nM, 100 nM) to screen the effective drug concentration of BRU in HGC-27 cells (**Figure 6A, C-E**). As previously reported, BRU was able to inhibit the protein expression of Nrf2, HO-1 and VEGF in a concentration-dependent manner. There was no significance between the Control group and the DMSO group (*P*>0.05). Compared with the DMSO group, the difference was not statistically significant at the concentration of 10 nM in the expression of VEGF (*P*>0.05), and the differences at the concentration of 20 nM, 40 nM, 80 nM and 100 nM were statistically significant (*P*<0.05). We selected 40 nM as the effective drug concentration of BRU for subsequent studies from the above results.

**Figure 5:**
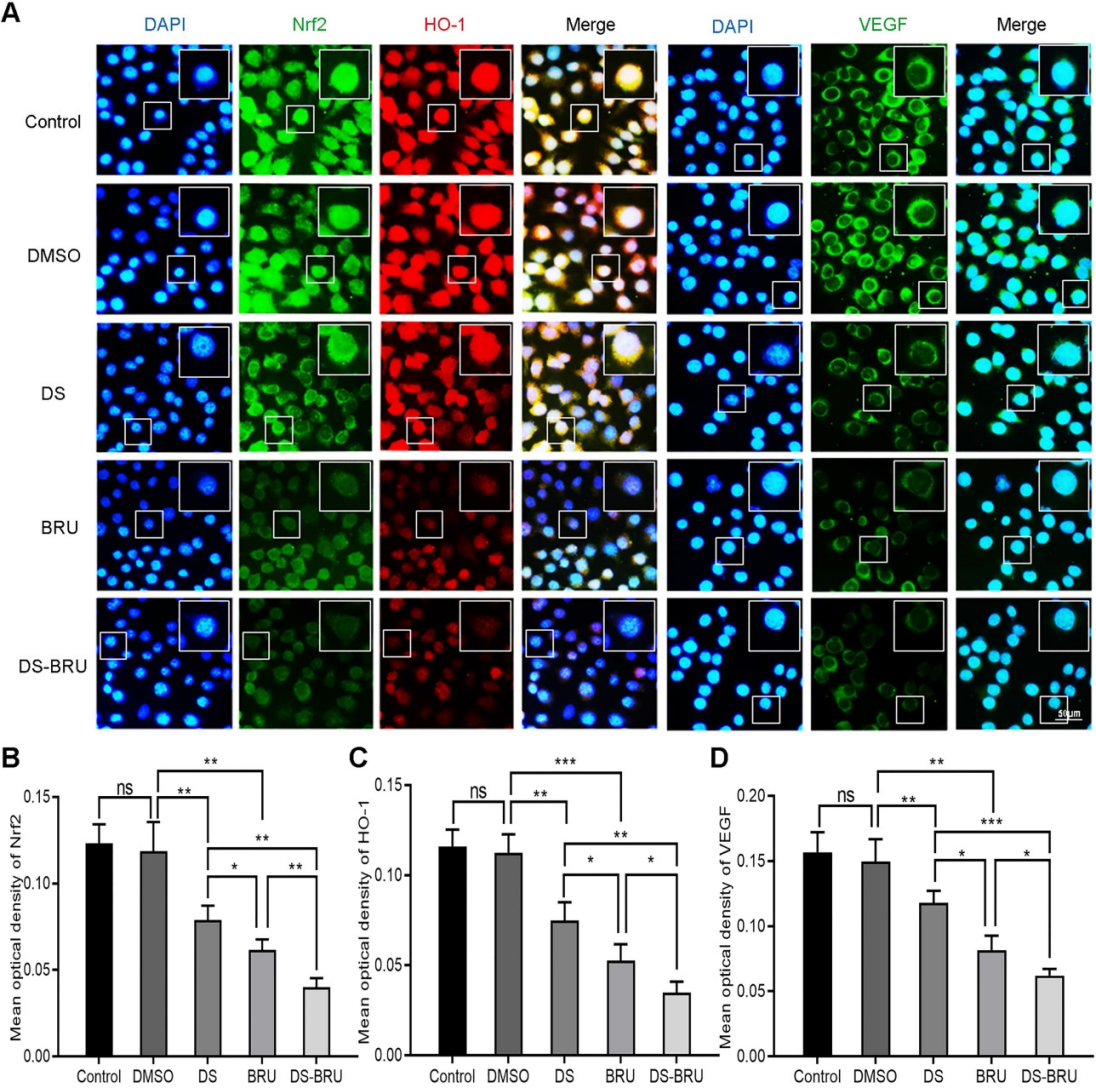
Immunofluorescence observes the effects of DS, BRU and their combination on the expression of Nrf2, HO-1 and VEGF in HGC-27 cells under hypoxic conditions. HGC-27 cells were treated with untreated, 0.1%DMSO, 0.3%DS, 40 nM BRU and 0.3%DS + 40 nM BRU for 24 h, respectively. Nrf2 and VEGF are labeled with FITC (green) while HO-1 is labeled with Rhodamine (red). Scale bar, 50 µm. ns, no significance. * *P* <0.05, ** *P* <0.01, *** *P* < 0.001.

Subsequently, immunofluorescence observed the effects of DS, BRU and their combination on the expression of Nrf2, HO-1 and VEGF in HGC-27 cells under hypoxic microenvironment (**Figure 5**). The fluorescence intensities of Nrf2, HO-1 and VEGF were significantly reduced in the DS group. However, the fluorescence intensities of Nrf2, HO-1 and VEGF in the BRU group decreased much more than in the DS group. Compared with the DS group or the BRU group, the degree of decrease in the fluorescence intensities of Nrf2, HO-1 and VEGF in the DS-BRU combination group was the most significant. This may be due to the pharmacological synergy between DS and BRU.

**Figure 6:**
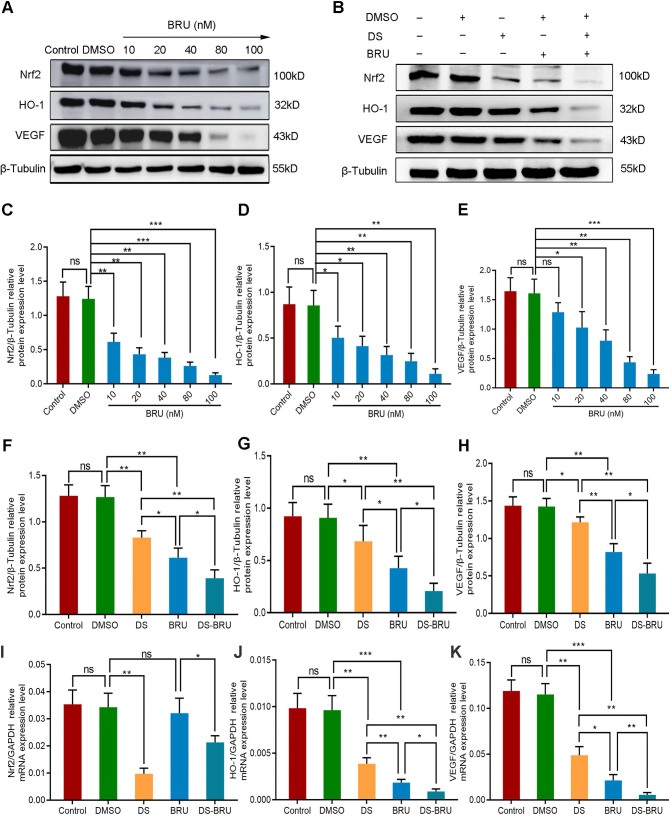
Western blot (A-H) and qPCR (I-K) analyze the effects of DS, BRU and their combination on the protein and mRNA expression of Nrf2, HO-1 and VEGF in HGC-27 cells under hypoxic conditions. (A,C-E) HGC-27 cells were treated with a gradient concentration of BRU (0 nM,10 nM, 20 nM, 40 nM, 80 nM, 100 nM) for 24 h. (B,F-K) HGC-27 cells were treated with untreated, 0.1% DMSO, 0.3% DS, 40 nM BRU and 0.3% DS + 40 nM BRU for 24 h, respectively. ns, no significance. * *P* <0.05, ** *P* <0.01, *** *P* < 0.001.

Thus, we evaluated the effects of DS, BRU and their combination on the protein and mRNA expression of Nrf2, HO-1 and VEGF in HGC-27 cells furtherly under the same conditions (**Figure 6B, F-K**). Expectedly, DS treatment markedly suppressed the protein levels of Nrf2, HO-1 and VEGF. BRU was able to inhibit the protein levels of Nrf2, HO-1 and VEGF. Its effect was more significant than DS. The inhibitory effect of DS and BRU co-treatment was greater than that of DS or BRU treatment alone. Similarly, DS also decreased the mRNA levels of Nrf2, HO-1 and VEGF. Interestingly, we noted that BRU did not significantly inhibit the mRNA level of Nrf2 but could inhibit the mRNA level of downstream genes of Nrf2. BRU may be a post-transcriptional regulator of Nrf2. Likewise, for the mRNA expression of downstream genes of Nrf2, the inhibitory effect of co-treatment was greater than that single treatment of DS or BRU.

Moreover, we used tube formation assay to evaluate the effect of down-regulation of Nrf2/HO-1 signaling pathway on the angiogenic potential of HGC-27 cells under hypoxic microenvironment (**Figure 9A, B**). DS treatment significantly reduced the number of tube formation. The decrease of tube formation in the BRU treatment group was more pronounced. As expected, there seems to be a synergistic effect in the co-treatment of DS and BRU.

#### Up-regulation of Nrf2/HO-1 signaling pathway increased VEGF expression and angiogenic potential in HGC-27 cells

We introduced the Nrf2 inducer tBHQ so as to compare the effects of up-regulation and down-regulation of Nrf2/HO-1 signaling pathway on VEGF expression and angiogenesis potential in HGC-27 cells. Firstly, western blot detected the protein expression of Nrf2, HO-1 and VEGF with a gradient concentration of tBHQ (0 μM, 2.5 μM, 5 μM, 10 μM, 20 μM, 50 μM) in HGC-27 cells (**Figure 8A, C-E**). As previously reported, tBHQ increased the protein expression of Nrf2, HO-1 and VEGF in a concentration-dependent manner. There was no significance between the Control group and the DMSO group (*P*>0.05). Compared with the DMSO group, the differences were not statistically significant at the concentration of 2.5 μM and 5 μM in the expression of HO-1 (*P*>0.05), and the differences at the concentration of 10 μM, 20 μM and 50 μM were statistically significant (*P*<0.01). We selected 10 μM as the effective drug concentration of tBHQ for subsequent studies from the above results.

**Figure 7:**
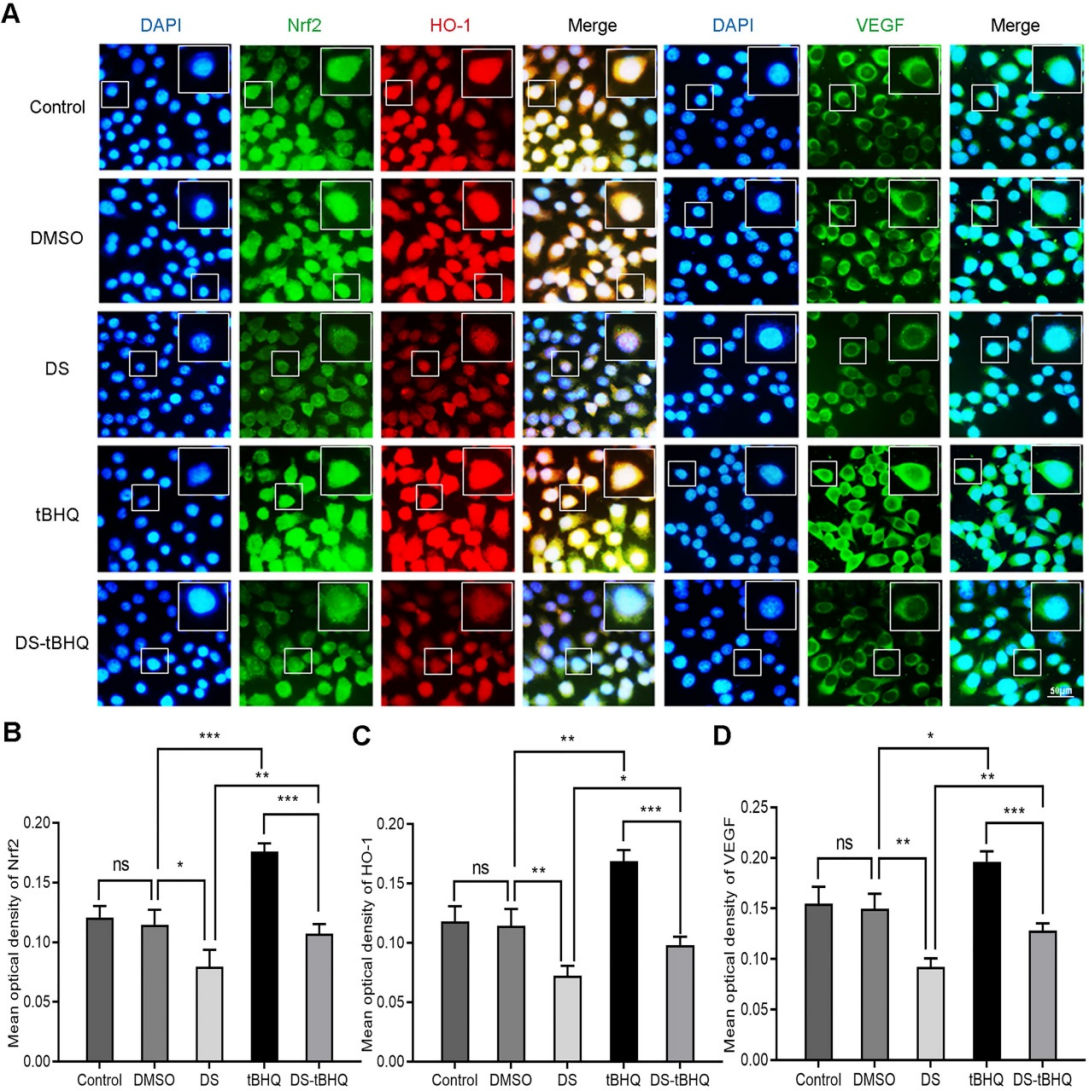
Immunofluorescence observes the effects of DS, tBHQ and their combination on the expression of Nrf2, HO-1 and VEGF in HGC-27 cells under hypoxic conditions. HGC-27 cells were treated with untreated, 0.1% DMSO, 0.3% DS, 10 µM tBHQ and 0.3% DS + 10 µM tBHQ for 24 h, respectively. Nrf2 and VEGF are labeled with FITC (green) while HO-1 is labeled with Rhodamine (red). Scale bar, 50 µm. ns, no significance. * *P* <0.05, ** *P* <0.01, *** *P* < 0.001.

Subsequently, Immunofluorescence observed the effects of DS, BRU and their combination on the expression of Nrf2, HO-1 and VEGF in HGC-27 cells under hypoxic microenvironment (**Figure 7**). DS still stably inhibited the fluorescence intensities of Nrf2, HO-1 and VEGF. The fluorescence intensity of the tBHQ group was significantly enhanced as expected. However, after co-treatment with DS, the fluorescence intensities of Nrf2, HO-1 and VEGF decreased.

**Figure 8:**
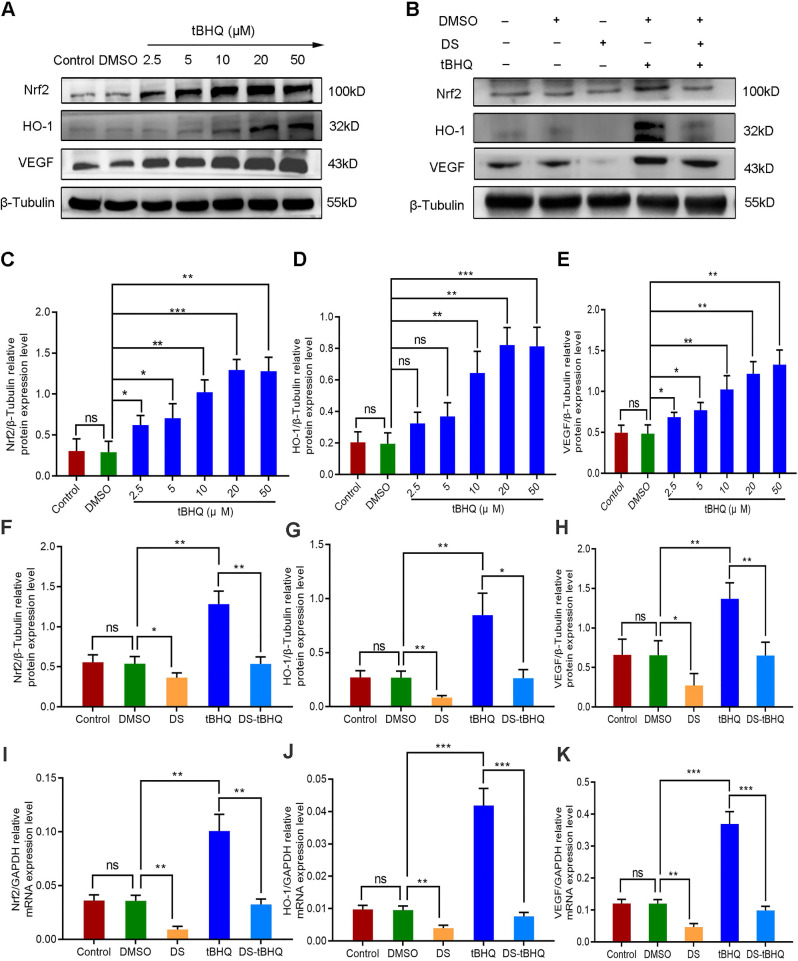
Western blot (A-H) and qPCR (I-K) analyze the effects of DS, tBHQ and their combination on the protein and mRNA expression of Nrf2, HO-1 and VEGF in HGC-27 cells under hypoxic conditions. (A,C-E) HGC-27 cells were treated with a gradient concentration of tBHQ (0 µM, 2.5 µM, 5 µM, 10 µM, 20 µM, 50 µM) for 24 h. (B,F-K) HGC-27 cells were treated with untreated, 0.1% DMSO, 0.3% DS,10 µM tBHQ and 0.3% DS + 10µM tBHQ for 24 h, respectively. ns, no significance. * *P* <0.05, ** *P* <0.01, *** *P* < 0.001.

Therefore, we evaluated the effects of DS, tBHQ and their combination on the protein and mRNA expression of Nrf2, HO-1 and VEGF in HGC-27 cells furtherly under the same conditions (**Figure 8B, F-K**). Expectedly, DS treatment markedly suppressed the protein and mRNA levels of Nrf2, HO-1 and VEGF. Meanwhile, tBHQ could significantly increase the protein and mRNA expression levels of Nrf2, HO-1 and VEGF. The protein and mRNA expression levels of Nrf2, HO-1 and VEGF decreased after co-treatment of DS and tBHQ. This may be due to the fact that DS has a certain antagonistic effect on tBHQ-induced Nrf2, HO-1 and VEGF expression enhancement.

**Figure 9:**
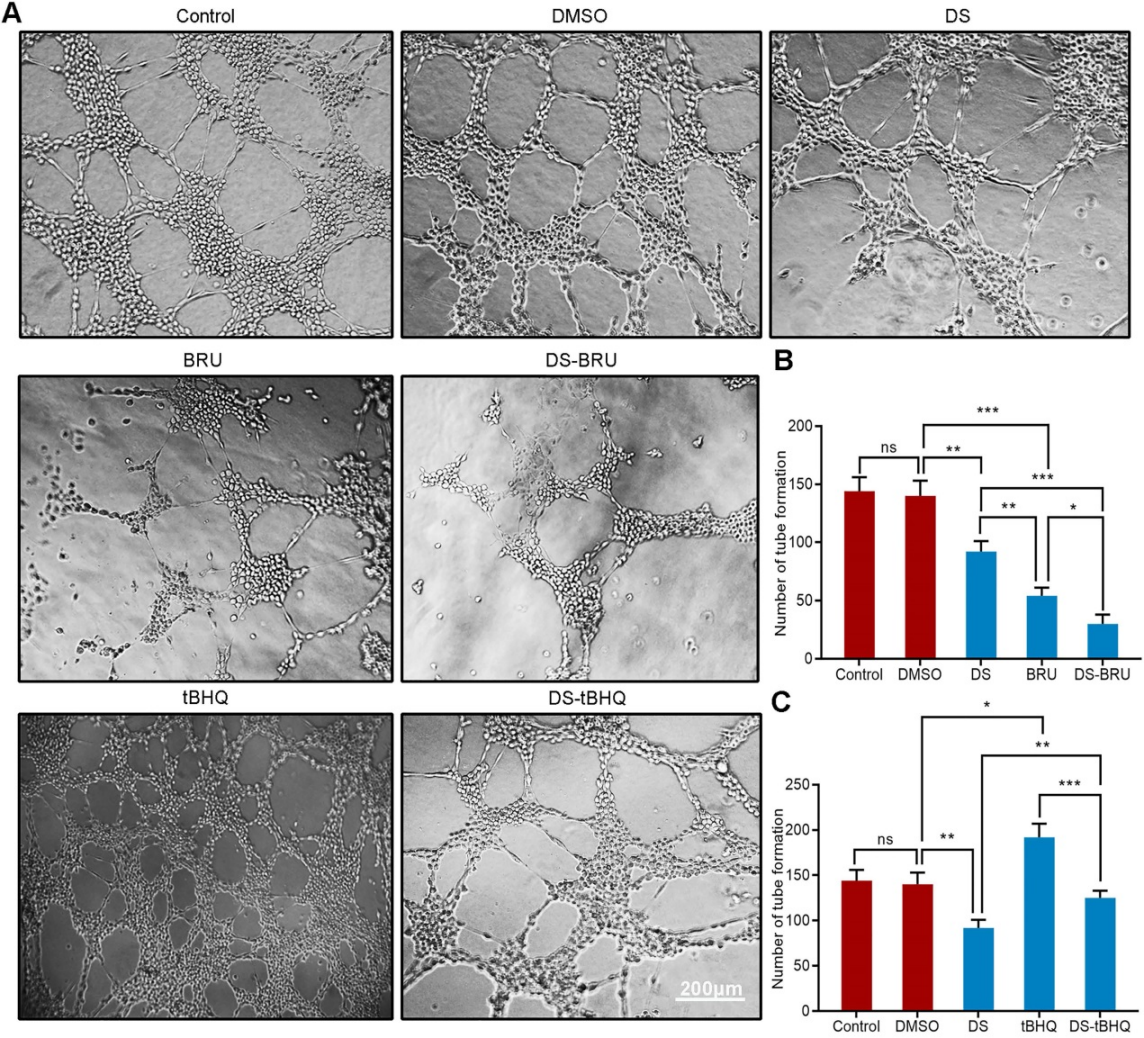
Tube formation assay shows the effects of BRU and tBHQ alone or in combination with DS on the angiogenic potential of HGC-27 cells under hypoxic conditions. HGC-27 cells were treated with untreated, 0.1% DMSO, 0.3% DS, 40 nM BRU, 0.3% DS + 40 nM BRU, 10 µM tBHQ, 0.3% DS + 10 µM tBHQ, accordingly. Scale bar, 200 µm. ns, no significance. * *P* <0.05, ** *P* <0.01, *** *P* < 0.001.

Similarly, tube formation assay evaluated the effect of down-regulation of Nrf2/HO-1 signaling pathway on the angiogenic potential of HGC-27 cells under hypoxic microenvironment (**Figure 9A,C**). The number of tube formation in the tBHQ group increased significantly. As expected, DS antagonized the tBHQ-induced increase in the angiogenic potential of HGC-27 cells.

### *In vivo* results

#### DS reduces the expression of Nrf2, HO-1 and VEGF in gastric cancer tissues of nude mice

**Figure 10:**
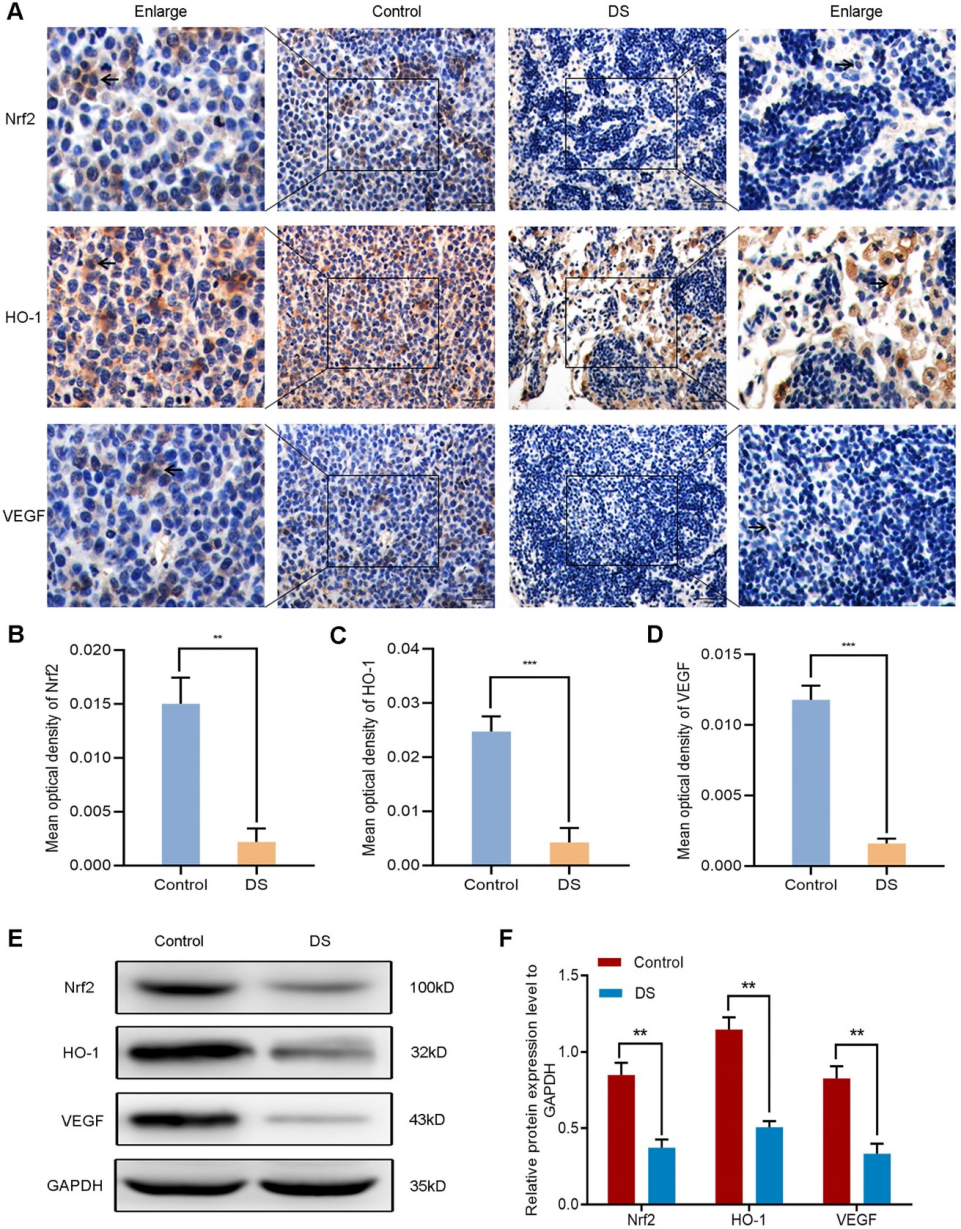
Immunohistochemical (A-D) and western blot (E-F) analyze the effects of DS on the expression of Nrf2, HO-1 and VEGF in tumor tissues of nude mice. Scale bar, 50 µm. ** *P* <0.01, *** *P* < 0.001.

The establishment of nude mouse intraperitoneal implantation metastasis model aimed to confirm whether DS could reduce angiogenesis via targeting the Nrf2/HO-1 signaling pathway. Based on our previous findings, DS could effectively reduce the number and volume of tumor nodules in nude mice. Here, we further explored the effect of DS on the Nrf2/HO-1 signaling pathway. Immunohistochemical observed the effects of DS on the expression of Nrf2, HO-1 and VEGF in gastric cancer tissues of nude mice (**Figure 10A-D**). The results showed that the positive expression of Nrf2, HO-1 and VEGF in DS group was significantly lower than that in Control group (*P*<0.01). Western blot analyzed the protein expression of Nrf2, HO-1 and VEGF in gastric cancer tissues of nude mice (**Figure 10E-F**). These results showed that the protein expression of Nrf2, HO-1 and VEGF in DS group was significantly lower than that in control group (*P*<0.01). In short, DS can effectively reduce the expression of Nrf2, HO-1 and VEGF in gastric cancer tissues of nude mice.

#### DS reduces the MVD in gastric cancer tissues of nude mice

**Figure 11:**
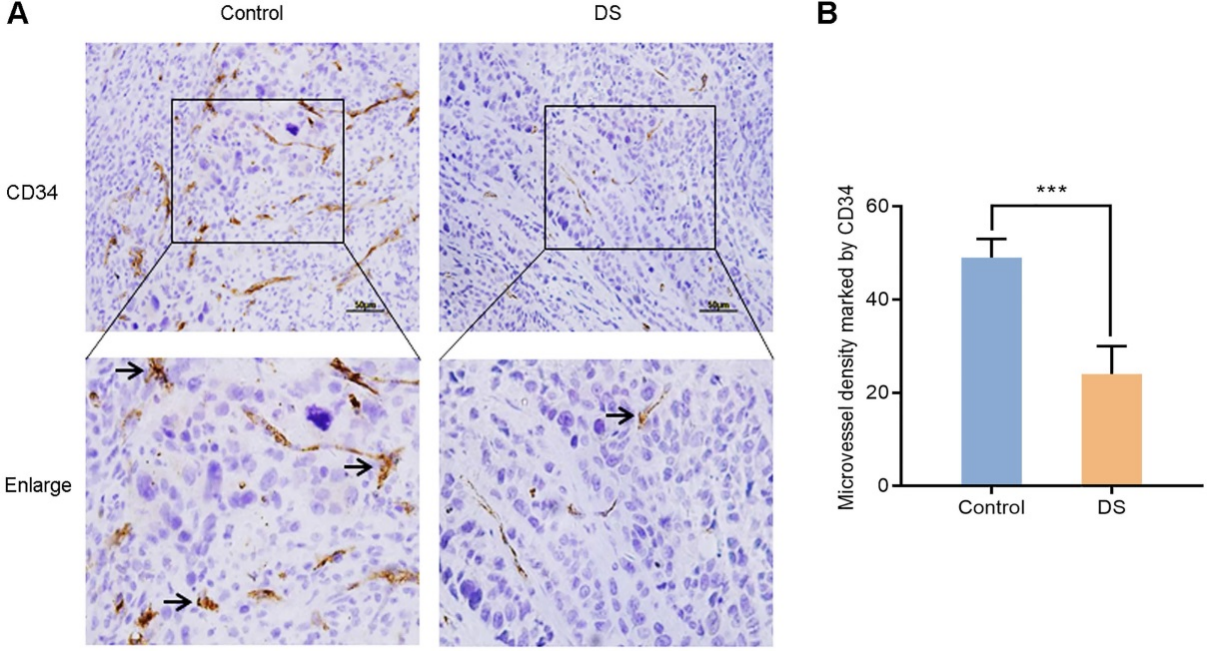
Immunohistochemical observes the effect of DS on MVD marked by CD34 in tumor tissues of nude mice. Scale bar, 50 µm. *** *P* < 0.001.

In order to further confirm the effect of DS on angiogenesis of gastric cancer tissues in nude mice, we compared the MVD of control group and DS treatment group in gastric cancer tissues of nude mice. CD34 was used to label vascular endothelial cells to reflect the MVD in the tumor tissue of nude mice to assess tumor angiogenesis. After labeling vascular endothelial cells with CD34, we found that CD34 was located in the cytoplasm of vascular endothelial cells (**Figure 11**). And we observed that the MVD of the DS group was significantly lower than that of the Control group (*P* <0.001).

#### The expression of Nrf2, HO-1 and VEGF in paracancerous tissue and gastric cancer tissues

**Figure 12:**
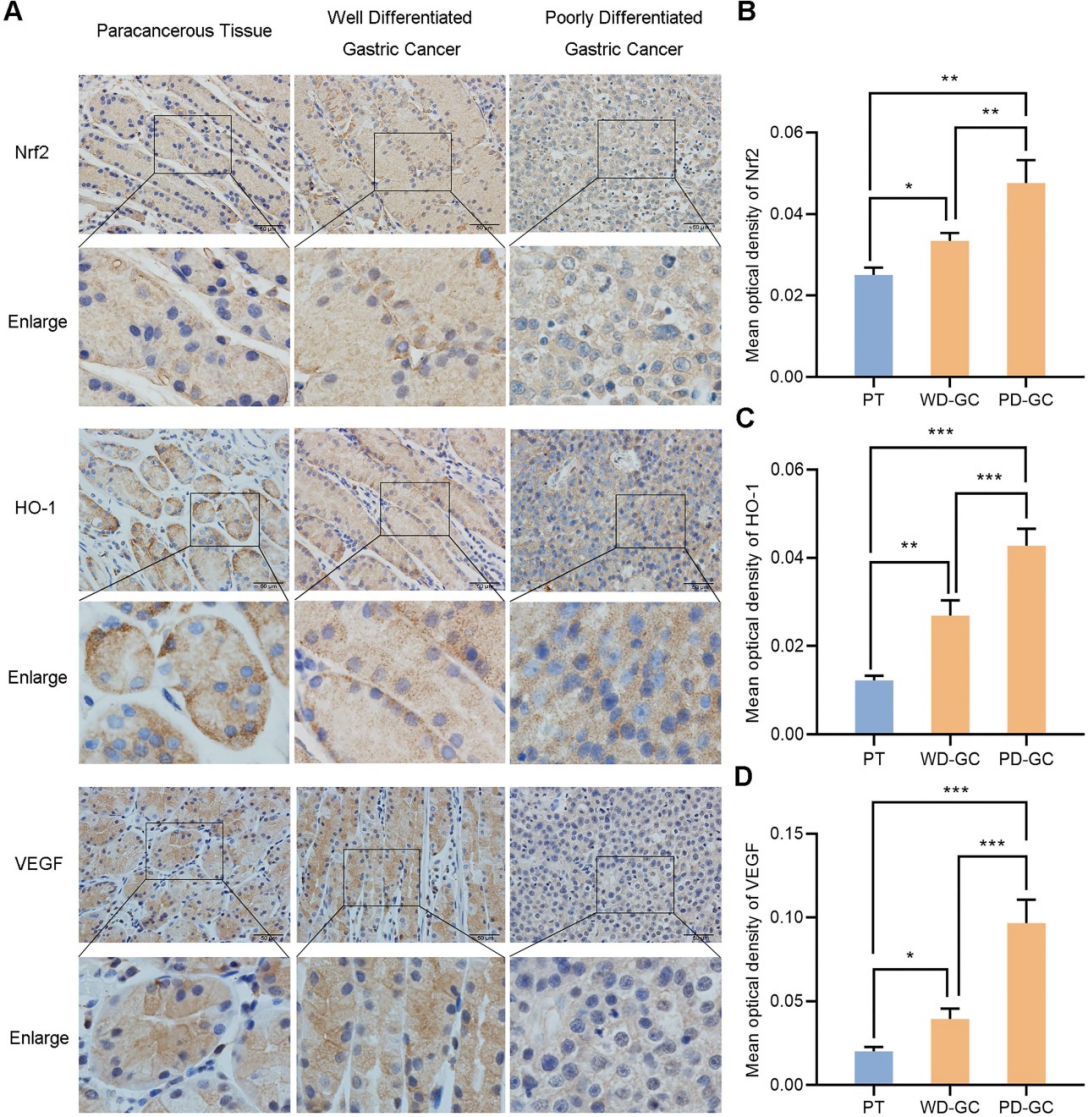
Immunohistochemical observes the expression of Nrf2, HO-1, VEGF in human paracancerous tissue and well, poorly differentiated gastric cancer tissues. Scale bar, 50 µm. * *P* <0.05, ** *P* <0.01, *** *P* < 0.001. PT, paracancerous tissue. WD-GC, well differentiated gastric cancer. PD-GC, poorly differentiated gastric cancer.

We selected the samples of human paracancerous tissue, well and poorly differentiated gastric cancer so as to observe the actual expression of Nrf2, HO-1 and VEGF in human gastric cancer tissues. Immunohistochemistry observed the expression of Nrf2, HO-1 and VEGF in the three groups of samples (**Figure 12**). Nrf2, HO-1 and VEGF showed low expression in paracancerous tissue but high expression in gastric cancer tissues. Nrf2, HO-1 and VEGF expression were weak in well differentiated gastric cancer tissues but strong in poorly differentiated gastric cancer tissues, respectively.

#### The MVD in paracancerous tissue and gastric cancer tissues

**Figure 13:**
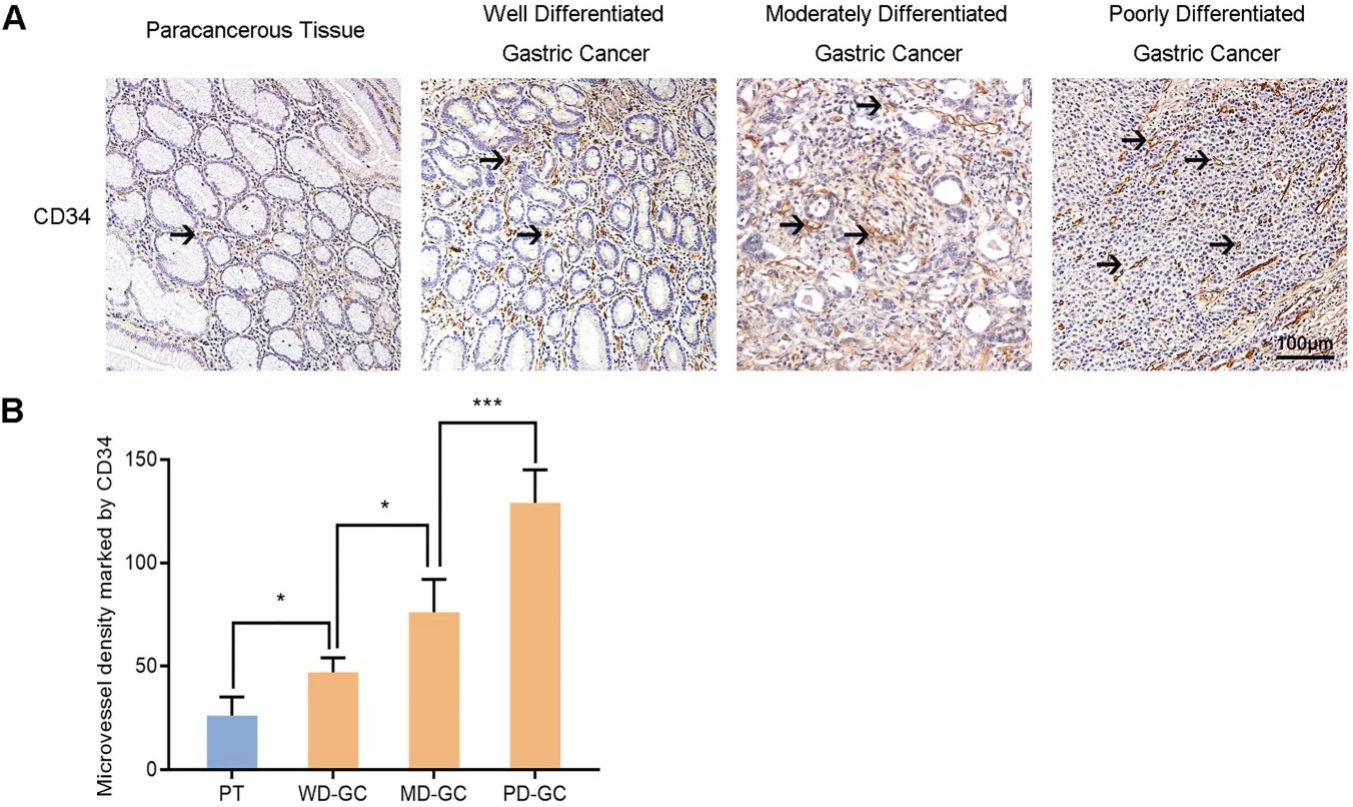
Immunohistochemical observes MVD marked by CD34 in human paracancerous tissue and well, moderately, poorly differentiated gastric cancer tissues. Scale bar, 100 µm. * *P* <0.05, *** *P* < 0.001. PT, paracancerous tissue. WD-GC, well differentiated gastric cancer. MD-GC, moderately differentiated gastric cancer. PD-GC, poorly differentiated gastric cancer.

Furthermore, we also selected the samples of human paracancerous tissue and well, moderately, poorly differentiated gastric cancer to explore the association between Nrf2/HO-1 signaling pathway and angiogenesis. MVD labeled with CD34 was used to assess tumor angiogenesis in the same way. Immunohistochemistry observed the expression of Nrf2, HO-1 and VEGF in the four groups of samples (**Figure 13**). The MVD showed low level in paracancerous tissue but high level in gastric cancer tissues. The MVD levels were low, moderate and high in well, moderately and poorly differentiated gastric cancer tissues, respectively.

## Discussion

To find novel and specific molecular markers can facilitate early detection and predict recurrence for gastric cancer. Nrf2 is the most active transcriptional regulator in CNC (cap 'n' collar) transcription factor family members 11. Under oxidative stress, Nrf2 can recognize and unite the antioxidant response element (ARE) or electrophilic response element (EpRE) to initiate the downstream transcription of multiple protective genes encoding antioxidant, anti-inflammatory and anti-apoptotic functions 12. Among them, HO-1 is the main effector of Nrf2-dependent expression 13. HO-1 is the initial enzyme and rate-limiting enzyme in the process of heme metabolism which catalyzes the degradation of heme to produce bilirubin, carbon monoxide (CO) and free iron (Fe^2+^). Among them, the degradation product CO of HO-1 can induce VEGF synthesis to stimulate angiogenesis, suggesting that HO-1 is a potential angiogenesis regulator 14.

The past studies show that Nrf2/HO-1 signaling pathway is one of the most important anti-oxidative stress mechanisms in cells, representing the maintenance of intracellular homeostasis and the adaptability of cellular stress responses 15. However, more and more studies are beginning to expose the dark side of Nrf2/HO-1 signaling pathway 16. Recent studies have shown that Nrf2 and HO-1 are found to be up-regulated in many different types of tumors and are closely related to tumor pathological angiogenesis. In rat cardiac microvascular endothelial cells, Nrf2 knockdown significantly decreases HO-1 and VEGF expression and tube formation 17. In human microvascular endothelial cells, knocking down of HO-1 decreases VEGF expression and tube formation 18. Depletion of Nrf2 causes reduction of tube formation through regulation of VEGF expression in glioblastoma 19, colon cancer 20 and bladder cancer 21 xenograft models. In addition, ZnPPIX (a HO-1 inhibitor) suppresses gastric cancer metastasis of tumor-bearing mice through down-regulation of HO-1 expression to reduce angiogenesis 22. At present, the role of Nrf2/HO-1 signaling pathway activation in gastric cancer tumor angiogenesis is not clear. Therefore, related researches targeting Nrf2, HO-1 and VEGF have great significance to anti-angiogenesis targeted therapy of gastric cancer.

VEGF is the strongest angiogenesis regulator wild known so far 23. VEGF is involved in the growth and metastasis of a variety of tumors by promoting angiogenesis, so it is considered as a basic therapeutic target for tumors 24. Numerous studies have confirmed that the growth, metastasis and prognosis of gastric cancer are related to angiogenesis 24. Interfering or blocking any part of VEGF and its signal pathway can effectively inhibit tumor angiogenesis, thereby suppressing tumor growth and metastasis.

In our previous *in vitro* studies, we have found that DS suppressed adhesion, proliferation, invasion, migration and epithelial-mesenchymal transition (EMT) but promoted apoptosis in human gastric cancer cells 10,25. These results indicate the promising potential of DS in the treatment of gastric cancer abdominal metastasis. *In vivo* studies have found that DS reduced the number and volume of tumor nodules in nude mice with gastric cancer 10. Angiogenesis is the core event of solid tumor growth and metastasis in the hypoxic microenvironment 3. Is the pharmacological effect of DS on tumor nodules in tumor-bearing mice achieved by suppressing angiogenesis? The effect of DS in angiogenesis of gastric cancer and its possible molecular mechanism would be the focus of this study. In this study, we investigated the association of Nrf2/HO-1 signaling pathway with VEGF and angiogenesis and whether DS could down-regulate VEGF to suppress angiogenesis by inhibiting Nrf2/HO-1 signaling pathway under hypoxic microenvironment in gastric cancer.

First of all, the effect of gradient concentration DS on the ability to induce angiogenesis in HGC-27 cells was tested by tube formation assay. The results showed that DS inhibited the ability of HGC-27 cells to induce angiogenesis in a concentration-dependent manner. It preliminary reveals that the functional mechanism by which DS reduces the number and volume of tumor nodules in nude mice in our previous research may be achieved by suppressing angiogenesis. In order to explore its possible molecular mechanism, we used immunofluorescence, western blot and qPCR to detect the effect of DS intervention gradient time (2, 8, 12 and 24 hours) on Nrf2, HO-1 and VEGF protein and mRNA expression. The results showed that the expression of Nrf2 and HO-1 at different time points showed an increasing trend with the prolongation of hypoxic incubation time, especially at 12 and 24 hours. This result indicates that hypoxia can induce the expression of Nrf2 and HO-1, and this expression is time-dependent. The expression levels of Nrf2 and HO-1 were positively correlated with the hypoxic duration, reflecting the promotion effect of the hypoxic microenvironment of gastric cancer on their expression. Nrf2 and HO-1 were up-regulated in gastric cancer that was consistent with the results of Nrf2 and HO-1 being confirmed to be up-regulated in other types of tumors 26. Meanwhile, the expression of DS group was lower than that of Control group at the same time point. The results show that DS can act continuously and significantly inhibit the expression of Nrf2 and HO-1. DS may act on Nrf2, the upstream regulator of HO-1, and inhibit Nrf2-dependent HO-1 expression.

At the same time, we found that VEGF was consistent with the results of Nrf2 and HO-1. The changes in the expression trends of Nrf2, HO-1 and VEGF were almost synchronous, suggesting that Nrf2, HO-1 and VEGF are signal pathways located on the same axis and Nrf2 plays an important role in regulating HO-1 and VEGF expression. Nrf2 may act as an upstream regulatory factor and positively regulate the expression of HO-1 and VEGF. VEGF is a key regulator of angiogenesis and a basic target for tumor anti-angiogenesis. It also suggested that Nrf2/HO-1 signaling pathway may be closely related to angiogenesis in human gastric cancer cells. Under hypoxic microenvironment, the protein and mRNA levels of Nrf2, HO-1 and VEGF in HGC-27 cells were significantly increased, and they were significantly reduced at all time points after DS intervention and accompanied by reduced angiogenesis. The above results suggest that there is a positive regulatory association between Nrf2, HO-1 and VEGF and the association is close. Thus, we speculate that DS may act on Nrf2/HO-1 signaling pathway to regulate the expression of VEGF and affect the angiogenesis of human gastric cancer cells. However, the above results cannot completely rule out the possibility of DS acts on the upstream target of Nrf2/HO-1 signaling pathway or affects the angiogenesis of human gastric cancer HGC-27 cells through other signal pathways. Hence, whether there is a clear regulatory association between the three or not in human gastric cancer cells and whether DS can directly act on Nrf2 to regulate the expression of VEGF in human gastric cancer cells needs further research to confirm.

In order to clarify the role and mechanism of DS more deeply, we introduced BRU, an inhibitor of Nrf2, to further investigate the regulation and effect of DS on Nrf2/HO-1 signaling pathway. BRU has shown through multiple studies that it can selectively inhibit Nrf2 through Keap1-dependent ubiquitination and proteasome degradation or protein synthesis 27. Firstly, for the purpose to verify the effectiveness of BRU in human gastric cancer cells, we used BRU to treat with HGC-27 cells in a gradient concentration. Western blot was used to detect the protein expression of Nrf2 and its downstream target genes HO-1 and VEGF. The results showed that BRU inhibited the expression of Nrf2 in HGC-27 cells in a concentration-dependent manner accompanied by the reduction of HO-1 and VEGF. We selected 40nM as the effective drug concentration of BRU from the above results for subsequent studies.Secondly, we used immunofluorescence, western blot, qPCR and tube formation assay to analyze the effects of DS, BRU and their combination on the expression of Nrf2, HO-1 and VEGF and the ability to induce angiogenesis in HGC-27 cells. The results showed that BRU reduced the protein levels of Nrf2 and its downstream genes and the mRNA level of Nrf2 downstream genes in HGC-27 cells at an effective concentration with the decrease in the number of angiogenesis. These results demonstrate that Nrf2/HO-1 signaling pathway positively regulates VEGF expression and angiogenesis in gastric cancer cells. Moreover, they also suggeste that BRU may affect the expression of downstream target genes HO-1 and VEGF by inhibiting the expression of Nrf2 in HGC-27 cells, thereby reducing the occurrence of blood vessels in gastric cancer cells. Similar results were observed by using DS under the same conditions. The effect of BRU was more significant than DS. Hence, we speculate that DS may also affect the expression of HO-1 and VEGF by inhibiting the expression of Nrf2 and thereby reducing the occurrence of gastric cancer blood vessels. In addition, we also observed an interesting phenomenon that the co-treatment of DS and BRU exerts a stronger antagonistic effect than DS or BRU treatment alone. It may be caused by the pharmacological synergy between DS and BRU. It provides us with a new idea, that is whether DS can not only target specific molecules to exert pharmacological effects to suppress gastric cancer angiogenesis, but also can be used in combination with certain specific molecular inhibitors to produce synergistic effects to make the inhibitory effect more attractive. This provides new possibilities for the combined use of DS in the development of anticancer drugs.

Furthermore, we tried to introduce the Nrf2 inducer tBHQ for comparative research. tBHQ is a specific Nrf2 inducer. It improves the stability of Nrf2, promotes nuclear translocation of Nrf2, increases the binding of Nrf2 to ARE, and then specifically activates the Nrf2-ARE signal pathway and its mediated transcription of downstream genes 28. We used a gradient concentration of tBHQ to treated with HGC-27 cells and found that tBHQ induced Nrf2 expression with its downstream target genes HO-1 and VEGF in a concentration-dependent manner in HGC-27 cells. We selected 10 μM as the effective drug concentration of tBHQ from the above results for subsequent studies. Likewise, we used DS, tBHQ and their combination to treat with HGC-27 cells under the same conditions. Immunofluorescence, western blot, qPCR and tube formation assay were used to analyze the expression of Nrf2, HO-1 and VEGF and the ability to induce angiogenesis. The results showed that the effective concentration of tBHQ could increase the protein and mRNA expression of Nrf2, HO-1 and VEGF with the increase in the number of angiogenesis. This was consistent with what we previously observed in BRU. This result re-emphasizes the importance of Nrf2/HO-1 signaling pathway for VEGF expression and angiogenesis in gastric cancer cells. In addition, after the combined use of DS and tBHQ, the protein and mRNA expression of Nrf2, HO-1 and VEGF were significantly reduced, accompanied by a decrease in the number of angiogenesis. It suggests that DS can effectively antagonize the induction of tBHQ on the Nrf2/HO-1/VEGF signal pathway. *In vitro* studies have shown that the activation of Nrf2/HO-1 signaling pathway may positively regulate angiogenesis of HGC-27 cells by mediating VEGF expression. DS may down-regulate VEGF by inhibiting Nrf2/HO-1 signaling pathway to suppress the angiogenesis of HGC-27 cells.

In order to verify our hypothesis, we constructed a nude mouse intraperitoneal implantation metastasis model. Immunohistochemistry and western blot were used to investigate the effects of DS on the expression of Nrf2, HO-1 and VEGF in gastric cancer tissues of nude mice. DS effectively reduced the protein expression of Nrf2, HO-1 and VEGF in gastric cancer tissues of nude mice. These results were consistent with the *in vitro* studies. It is well known that MVD is an important indicator to assess tumor angiogenesis. CD34 is one of the most common vascular endothelial cell markers 29. We used CD34 to label vascular endothelial cells to assess tumor angiogenesis. DS significantly reduced the MVD of gastric cancer tissues in nude mice. It suggested that DS can suppress gastric cancer angiogenesis in nude mice. We also collected specimens of paracancerous tissue and gastric cancer tissues with different degrees of differentiation. We then observed the expression of Nrf2, HO-1 and VEGF in different differentiation of gastric cancer tissues by immunohistochemistry. We found that Nrf2, HO-1 and VEGF showed low expression in paracancerous tissue but high expression in gastric cancer tissues. Nrf2/HO-1 signaling pathway may be involved in the occurrence and development of gastric cancer. Nrf2, HO-1 and VEGF expression were weak in well differentiated gastric cancer tissues but strong in poorly differentiated gastric cancer tissues. Nrf2/HO-1 signaling pathway may be related to the malignant degree of gastric cancer and poor prognosis. Furthermore, we detected CD34-labeled MVD values in paracancerous tissue and well, moderately, poorly differentiated gastric cancer tissues. The MVD showed low level in paracancerous tissue but high level in gastric cancer tissues. The MVD levels were low, moderate and high in well, moderately and poorly differentiated gastric cancer tissues, respectively. These results demonstrate that the MVD values of these tissues showed synchronism and correlation with the expression of Nrf2, HO-1 and VEGF. Nrf2/HO-1 signaling pathway may participate in the malignant process of gastric cancer by affecting angiogenesis. We discussed the possible mechanism that DS inhibited Nrf2/HO-1 signaling pathway. We observed that the nuclear expression of Nrf2 in the DS group seemed to be reduced, and DS could effectively reduce the mRNA level of Nrf2. It suggests that the DS may have potential to inhibit Nrf2 signal pathway by repressing both nuclear translocation and transcription of Nrf2. However, further systematic and molecular studies are required to understand intracellular dynamics in the DS effect.

In summary, Nrf2/HO-1 signaling pathway may positively regulates VEGF to promote angiogenesis in gastric cancer. The effect of DS on gastric cancer angiogenesis may be through the inhibition of Nrf2/HO-1 signaling pathway under hypoxic microenvironment. The functional mechanism is to inhibit Nrf2 to decreased its downstream target gene HO-1, and then further down-regulate VEGF to suppress gastric cancer angiogenesis, thereby affecting the growth and metastasis of gastric cancer. This study is expected to provide a new molecular target for anti-angiogenesis treatment of gastric cancer and an important theoretical basis for the subsequent research and clinical application of DS.

## Abbreviations

DS: dextran sulfate
Nrf2: nuclear factor-erythroid 2-related factor 2
HO-1: heme oxygenase-1
VEGF: vascular endothelial growth factor
PBS: phosphate buffered saline
DMSO: dimethyl sulphoxide
BRU: brusatol
tBHQ: tert-butylhydroquinone
PT: paracancerous tissue
WD-GC: well differentiated gastric cancer
MD-GC: moderately differentiated gastric cancer
PD-GC: poorly differentiated gastric cancer
MVD: mircrovessel density
